# Compared with dietary behavior and physical activity risk, sedentary behavior risk is an important factor in overweight and obesity: evidence from a study of children and adolescents aged 13–18 years in Xinjiang, China

**DOI:** 10.1186/s12887-022-03646-y

**Published:** 2022-10-07

**Authors:** He Liu, Cunjian Bi, Hongniu Lin, Wei Ma, Jie Zhang, Yan-Yan Hu, Jing-Zhi Liu

**Affiliations:** 1 Research Department of Physical Education, Xinjiang University, Urumqi, 800002 China; 2 School of Physical Education, Chizhou University, Chizhou, 247000 China; 3 Sports Health Promotion Center, Chizhou University, Chizhou, 247000 China; 4 Xinjiang Sports Vocational and Technical College, Urumqi, 830007 China; 5 Research Department of Physical Education, Xinjiang Institute of Engineering, Urumqi, 830023 China

**Keywords:** Xinjiang China, Children and adolescents, Nutritional status, Eating behavior, Physical activity, Cross-sectional analysis

## Abstract

**Background:**

Malnutrition or insufficient physical activity (PA) is a risk factor for obesity and chronic disease in children and adolescents. Affected by different economic circumstance, ethnic, dietary behavior, physical activity and other factors, children and adolescents in Xinjiang, China are facing a severe situation of overweight and obesity prevention and control. It is necessary to analyze the dietary behavior and physical activity of children and adolescents with different nutritional status and the relationship between them.

**Methods:**

Using a stratified cluster sampling method in Xinjiang, China, 4833 middle school students aged 13–18 were selected., and tests for height and weight were conducted. Self-assessment questionnaires were completed for Dietary Behaviors, Physical Activity, and Sedentary Behaviors as well. Chi-square test, Logistic regression analysis and other methods were used to analyze the relationship between Dietary Behaviors, Physical Activity, Sedentary Behaviors and other health behavior risk factors and Weight and BMI.

**Results:**

Children and adolescents aged 13–18 in Xinjiang, China, girls had a lower BMI compared with boys(19.49 *VS*. 20.41). The proportions of Underweight, Overweight and Obese among girls were lower (Underweight: 11.8 *VS*. 14.5; Overweight: 7.6 *VS*. 12.7; Obese 2.3 *VS*. 7.0).Boys with higher risk of sedentary had a 1.46-fold higher risk of developing Overweight/Obese than those with lower risk of sedentary (95%*CI*: 1.07–2.01).Girls with higher risky diet had a 1.56-fold higher risk of developing Underweight than those with lower risky diet (95%*CI*: 1.11–2.19). For all participants, the risk of Overweight/Obese in children and adolescents with higher risk of sedentary was 1.45 times more than that of children and adolescents with lower Risk sedentary (95%CI: 1.12 ~ 1.88). Overall, Weight had a significant correlation with PA risk (*r* = 0.076, *P* < 0.01) and sedentary behavior risk (*r* = 0.035, *P* < 0.05). BMI had a key correlation with PA risk (*r* = 0.064, *P* < 0.01) and sedentary behavior risk (*r* = 0.037, *P* < 0.05).

**Conclusions:**

The detection rate of Underweight among children and adolescents aged 13–18 in Xinjiang, China is higher, while the detection rate of Overweight and Obese is lower than that of the whole country. Static behavior was an important factor affecting the occurrence of Overweight and Obese in children and adolescents, and the performance of boys was more obvious than that of girls.The results further improve the data on the weight status of Chinese children and adolescents and their influencing factors, and call on Chinese society and schools to continue their efforts to prevent and reduce malnutrition and obesity among children and adolescents in Xinjiang.

## Introduction

Meta-analyses have suggested that long-term healthy dietary habits and adequate PA can not only promote human health and the body's immune capacity but can also reduce the incidence and all-cause mortality of obesity, cardiovascular disease, type 2 diabetes, and cancer [1–3]. Overweight and obesity have become serious public health problems in recent years, and childhood obesity increases the odds of problems such as obesity, early death, and disability in adulthood. As of April 2020, more than 340 million children and adolescents aged 5 and 19 years worldwide were overweight or obese, and the prevalence of overweight or obesity in this age group increased sharply from 4% in 1975 to more than 18% in 2016. In 2020, statistics showed that the proportion of obese or overweight children and adolescents in the United States was 41% [4]. In China, the detection rate of overweight and obesity among school children aged 6–17 increased year by year and reached 20% in 2020 [5] and is expected to be 28.0% in 2030 [6].

A proper diet contributes to health, and one reason for the high incidence of obesity and overweight is the increased intake of energy-rich foods, such as sugar and fat, including among children and adolescents. Nearly half (46.7%) of children and adolescents aged 2–18 years in Australia consume drink sugary drinks [7]. In China, the consumption rate of sugary milk drinks and sugar-sweetened beverages among children and adolescents is more than 30% and 25%, respectively, which is significantly higher than the rates in adults [8]. The WHO meta-analysis of 5 prospective cohort studies in children showed that after 1 year of follow-up, higher SSB intake was associated with a 55% higher risk of overweight/obesity than those with the lowest intake. In free-living, free-eating populations, free sugar or ssb intake is associated with body weight [9].A high intake of fruit juices and soft drinks contributes to excessive weight gain and obesity in children.Recent research has found a positive association between regular SSBs consumption and weight gain [10]. The World Health Organization (WHO) recommends increasing the daily intake of fruits, vegetables, legumes, whole grains and nuts; however, in 2014, intakes among European children and adolescents aged 12.5–17.5 years were only 50% of recommended levels, and intake of and milk and dairy products was 66.6% [11]. From 2013 to 2016, nearly 90% of people in the United States did not reach the recommended standard for vegetable and milk intake; average daily intake of fruits and vegetables among children aged 14–18 years was 50% of the recommended intake, and milk intake was approximately 65% of recommendations [4]. In terms of the effect of dairy consumption on growth and development in children aged 6–18 years, it has been shown that those who consume sufficient milk and dairy products are more likely to have a lean body composition [12]. Between 2016 and 2017, the consumption rate of milk and dairy products among Chinese middle school students was 82.5%; only 44.1% consumed milk and dairy products daily, and only 20.4% of these students reached the intake level recommended in the Dietary Guidelines for Chinese Residents (2016) [13]. The average intake of milk and dairy products among Chinese residents has been at a low level, but the consumption rates among children and adolescents are higher than rates among adults [8]. In 2019, the per capita milk consumption in Xinjiang Uygur Autonomous Region was 21.1 kg, which is much higher than the national per capita consumption of 12.5 kg, ranking third in China after in Beijing and Inner Mongolia [14].

In 2009, physical inactivity was identified as the fourth leading risk factor for NCDs, causing more than 3 million preventable deaths.[]Current estimation of global physical inactivity suggests that 27.5% of adults and over 80% of adolescents aged 11–17 years do not meet the existing WHO recommendations for physical exercise to promote health [15–17]. In the past 20 years, the overall PA among Chinese residents has shown a declining trend each year. In particular, the PA level of children and adolescents is generally insufficient. The average time engaged in medium- and high-intensity PA among Chinese children and adolescents is 37.66 min/d, which is far lower than these times in Canada (59 min/d), the United Kingdom (60.5 min/d), and the United States (48 min/d) [18]. An objective and subjective measure of physical activity in the United States gave qualitatively similar results in terms of gender and age activity patterns: 42% of children aged 6–11 received the recommended minimum daily amount of 60 min of physical activity, while only 8% of Teens achieve this. Among adults, 30 min of daily physical activity is less than 5% [19]. For economical, equipment, and technical reasons, we chose a self-reported method to assess physical activity.With the development of electronic technologies, digital media devices have increasingly become the main cause of reduced PA in children and adolescents. Among Australian children and young adults, only 30% of those aged 5 to 17 years meet the guideline for daily screen-based sedentary behavior [20]. Screen overuse in children and adolescents is associated with physical and mental health risks [21]. In 2016, the prevalence of obesity among Chinese children and adolescents aged 9–17 years was approximately 12%, and around 37% used a screen for more than 2 h per day [22]. Epidemiological studies have shown that children who use more screen media also eat less fruits and vegetables, consume more high-energy snacks, high-energy drinks and fast food, and consume more calories per hour [23]. During the COVID-19 pandemic, nearly 80% of children and adolescents in China significantly increased their daily screen use time, with 42.3%–48.2% of students using digital devices for more than 5 h a day to study online, especially high school students [24]. This means a significantly increased risk of diseases such as obesity owing to screen overuse. The COVID-19 pandemic has accelerated the evolution of this trend. Both the physical quality index (body mass index, BMI) and obesity increased owing to the decline in PA levels during the pandemic [25, 26].

Xinjiang Uygur Autonomous Region is located at the northwest border of China. There are 13 ethnic groups in Xinjiang, including Uygur, Han, Kazak, and Hui. The special regional environment in Xinjiang results in a variety of eating habits. The diet mainly comprises wheat, beef, mutton, dairy products, and nuts. According to China's 2020 Obesity Prevention and Control Implementation Plan for Children and Adolescents, Xinjiang is listed as a middle-level area of overweight and obesity prevalence among children and adolescents. In view of the severe situation facing children and adolescents in terms of overweight and obesity, we conducted an investigation among 5044 children and adolescents aged 13–18 years in Xinjiang, China to analyze the dietary behavior and PA of children and adolescents with different nutritional status and the relationship between them. We hypothesized that both dietary behavior and physical activity could influence the nutritional status of children and adolescents in Xinjiang, China. Our research will provide help for the physical and mental development of children and adolescents in Xinjiang, China. At the same time, it will also provide effective public health policies for local government departments and education administrative departments.

## Materials and methods

### Study design and participants

**Fig. 1:**
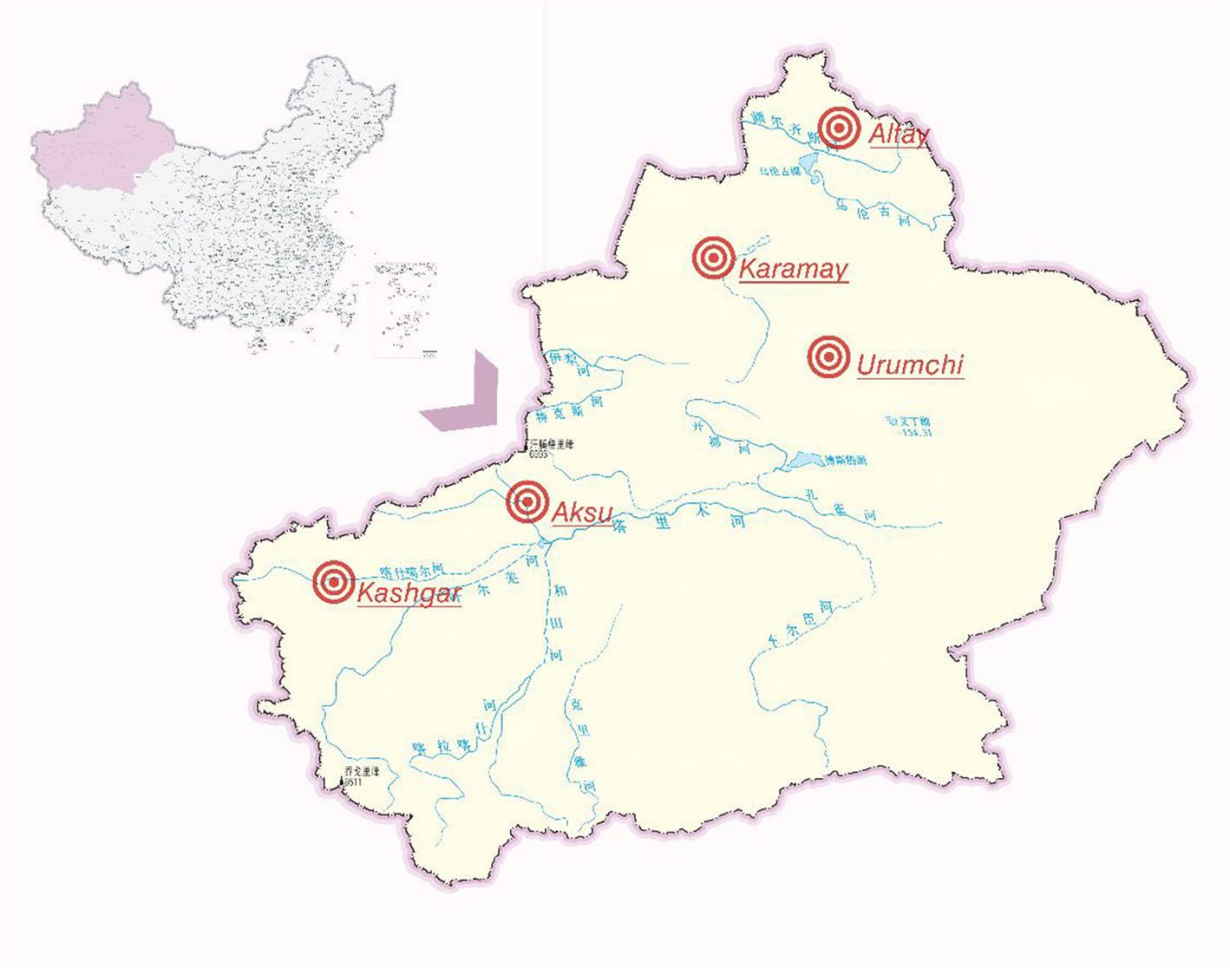
Schematic diagram of the study area in Xinjiang Uygur Autonomous Region

This study was conducted in Urumqi, Altay, Karamay, Aksu, and Kashgar, in Xinjiang, China from September to December 2021 (Fig. 1). Using a stratified cluster sampling method, two to four middle schools were selected in each region, and another two to three classes were randomly selected in each grade. In this study, 5044 middle school students aged 13–18 years were selected and asked to complete a questionnaire. After excluding 211 invalid questionnaires, 4833 valid questionnaires were finally collected; the effective recovery rate was 95.82%.

**Fig. 2:**
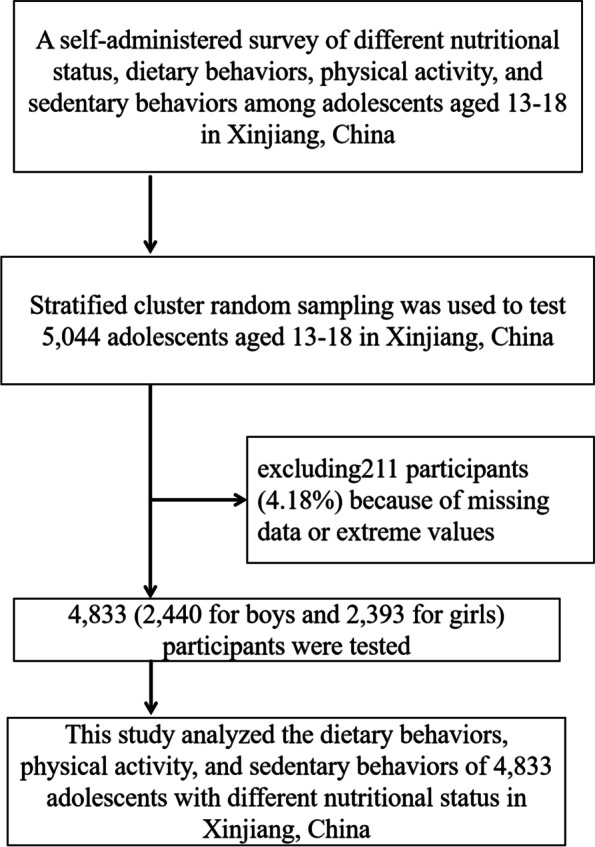
Participant selection process

This study belongs to the status quo investigation research, and the research index is enumeration data, so the sample estimation formula is:$n=\frac{Z{a/2}^{2}\times p\left(1-p\right)}{{d}^{2}}$, *d* = 0.15 × *p*, *α* = 0.05 (two-sided), = 1.96. The number of middle school students in Xinjiang is about 150,000, and the total population of Xinjiang is 25.89 million, accounting for 0.58%. Therefore, it is 0.58, and the calculated sample is about 60 people. Considering the loss rate of 10%, about 66 people need to be tested. This study tested a total of 5 regions in Xinjiang, 6 age groups, and urban and rural distribution. Therefore, this study should test a total of about 3960 people. This study used a class cluster sampling survey, and a total of 4833 valid data were investigated.See the selection process in Fig. 2.

Prior to the questionnaire survey, students, parents or legal guardians had obtained written informed consent. The study was conducted in accordance with the Declaration of Helsinki, and approved by the Human Subjects Protection Committee of Chizhou University (0236–2020).

### Measures

This study was part of the Key Laboratory of Adolescent Health Assessment and Exercise Intervention, Ministry of Education, East China Normal University research project. The study questionnaire was finalized with reference to the Chinese National Student Physical Health Survey questionnaire and the US Centers for Disease Control and Prevention Youth Risk Behavior Surveillance System questionnaire [27, 28]. The overall questionnaire included eight parts: basic information, physical exercise, eating habits, sleep status, health risk behavior, mental health, PA, and physical examination. Students were required to complete the total of 108 items within 20–30 min.The questionnaire was determined after expert discussion and analysis, and 202 middle school students were selected for pre-investigation at 15-day intervals, which indicated that the questionnaire had good reliability and validity, cronbach's α coefficient is 0.85.

This survey included six questions addressing sociodemographic characteristics (age, urban or rural residence, school type, living on campus, father's education, and mother's education), four questions addressing dietary behaviors (consumption of sugar-sweetened drinks, breakfast, dairy or soy milk, and eggs), six questions addressing physical activity (low intensity PA, moderate-to-vigorous PA [MVPA], after school PA, means of transportation to and from school, time from home to school, and muscle strength training), three questions on sedentary behaviors (sitting to study after school, watching TV, using a phone or tablet) and two anthropometric measures (height, weight).

Each student was required to choose a response to each item according to their own situation. With reference to previous studies and related behavior guidelines [29–32], the categories of dietary behaviors, PA, and sedentary behaviors were scored 0 point for healthy behaviors or 1 points for unhealthy behaviors, and the number of risk behaviors was calculated. The categories dietary behaviors, PA, and sedentary behaviors included four, six, and three risk behaviors, respectively. According to the median number of risk behaviors in each category, for dietary behaviors, ≤ 1 risk behavior was defined as “low dietary risk” and > 1 risk behavior was defined as “high dietary risk.” For PA, ≤ 2 risk behaviors were defined as “low PA risk” and > 2 risk behaviors was defined as “high PA risk.” Similarly, ≤ 1 sedentary risk behavior was defined as “low sedentary risk” and > 1 risk behavior was defined as “high sedentary risk.”

Students’ height and weight were measured according to the methods and instruments required in the national physical health test for Chinese students. Height was measured to the nearest 0.1 cm and weight to the nearest 0.1 kg [27]. BMI was calculated based on height and weight as weight (kg)/height (m)^2^. Chinese school-aged children were categorized as underweight, normal weight, overweight, or obese [33].

### Statistical analysis

Demographic characteristics are expressed as mean ± standard deviation or number and percentage. We used the *t*-test for comparisons of continuous variables by sex and the chi-square test for comparisons among PA, sedentary behavior, and dietary behavior by sex and nutritional status. We adjusted for sex, urban or rural residence, school type, father's education, mother's education, and living on campus in logistic regression analysis to analyze the effect of dietary behaviors, PA, and sedentary behaviors on body weight. The crude odds ratio (COR), adjusted odds ratio (AOR), and 95% confidence interval (CI) for children and adolescents with different nutritional statuses were calculated using GraphPad Prism 8 (GraphPad Software, San Diego, CA, USA). Correlation analysis of child and adolescent risk behaviors, body weight, and BMI by sex and school type was conducted using Pearson's correlation analysis. Statistical analysis of the data was performed using IBM SPSS 25.0 (IBM Corp., Armonk, NY, USA), and the significance level was set to *P* < 0.05.

## Results

### Participant characteristics

According to Table 1, 50.49% (2440/4833) of children aged 13–18 years in Xinjiang, China were male. Those in middle school accounted for 70.1% (3390/4833) of the study population. Compared with boys, girls had a lower BMI (19.49 VS. 20.41). The proportions of Underweight, Overweight and Obese among girls are all lower (Underweight: 11.8 VS. 14.5; Overweight: 7.6 VS. 12.7; Obese 2.3 VS. 7.0).

**Table 1:** Comparison of different demographic characteristics of children and adolescents aged 13–18 years in Xinjiang, China

Sort	n	Total(*n* = 4833)	Boy(*n* = 2440)	Girl(*n* = 2393)	*t* /*χ*^2^ Test	*p*-Value
Height(cm)	4833	165.22 ± 9.02	168.83 ± 9.61	161.54 ± 6.58	30.715	< 0.001
Weight(kg)	4833	54.83 ± 12.37	58.63 ± 14.09	50.96 ± 8.75	22.690	< 0.001
BMI(kg/m^2^)	4833	19.95 ± 3.45	20.41 ± 3.85	19.49 ± 2.91	9.368	< 0.001
**BMI categories**					118.563	< 0.001
Underweight	638	13.2	14.5	11.8		
Normal	3476	71.9	65.8	78.2		
Overweight	492	10.2	12.7	7.6		
Obese	227	4.7	7.0	2.3		
**Urban and rural**					0.401	0.526
Urban	2367	49.0	48.5	49.4		
Rural	2466	51.0	51.5	50.6		
**School type**					7.133	0.008
Middle school	3390	70.1	68.4	71.9		
High school	1443	29.9	31.6	28.1		
**Living on campus**						
No	2904	60.1	60.5	59.6	0.409	0.523
Yes	1929	39.9	39.5	40.4		
**Father's education**					1.907	0.592
Junior high and below	2535	52.5	53.1	51.8		
High school	723	15.0	14.9	15.0		
Junior college or above	999	20.7	19.9	21.4		
Unknown	576	11.9	12.1	11.7		
**Mother's education**					11.478	0.009
Junior high and below	2289	47.4	46.4	48.3		
High school	888	18.4	19.4	17.3		
Junior college or above	1141	23.6	22.5	24.8		
Unknown	515	10.7	11.7	9.6		

Data presented as mean ± standard deviation or number and percentage

*BMI* Body mass index

### Assessment of dietary behaviors, physical activity, and sedentary behaviors

Table 2 shows that for dietary behaviors, boys and girls showed differences in the frequency of weekly egg consumption (*χ*^2=^6.977, *P* < 0.05). Children and adolescents with different nutritional statuses had differences in the number of times per day sugar-sweetened drinks were consumed (*χ*^2=^45.641, *P* < 0.001) as well as differences in the weekly frequency of eating eggs (*χ*^2=^8.698, *P* < 0.05).

**Table 2:** Comparison of dietary behaviors, physical activities, and sedentary behaviors detection rates among children and adolescents by sex and nutritional status

Sort	Boy	Girl	Total	*χ*^2^ Test	*p*-Value	Underweight	Normal	Overweight	Obese	*χ*^2^ Test	*p*-Value
**Dietary Behaviors**											
**Sugar sweetened drinks**				2.443	0.118					45.641	< 0.001
< 1 time/d	27.1	26.1	26.1			33.4	26.5	18.9	15.0		
≥ 1 time/d	72.9	73.9	73.9			66.6	73.5	81.1	85.0		
**Breakfast**				0.001	0.981					3.269	0.352
< 1 d/wk	1.7	1.7	1.7			1.9	1.6	1.4	3.1		
≥ 1 d/wk	98.3	98.3	98.3			98.1	98.4	98.6	96.9		
**Dairy or soy milk**				0.035	0.852					5.888	0.117
< 1 d/wk	7.7	7.5	7.6			8.3	7.5	5.7	10.6		
≥ 1 d/wk	92.3	92.5	92.4			91.7	92.5	94.3	89.4		
**Eat eggs**				6.977	0.008					8.698	0.034
< 1 d/wk	10.2	12.6	11.4			8.5	11.9	10.6	14.5		
≥ 1 d/wk	89.8	87.4	88.6			91.5	88.1	89.4	85.5		
**Physical Activity**											
**Low intensity PA**				16.058	< 0.001					4.083	0.253
< 60 min/d	26.7	31.9	29.3			29.3	29.9	25.6	27.8		
≥ 60 min/d	73.3	68.1	70.7			70.7	70.1	74.4	72.2		
**Moderate-to-vigorous PA**				32.704	< 0.001					1.023	0.796
< 60 min/d	5.0	9.3	7.1			7.7	7.1	6.3	7.9		
≥ 60 min/d	95.0	90.7	92.9			92.3	92.9	93.7	92.1		
**After school PA**				1.274	0.259					94.472	< 0.001
< 60 min/d	80.5	79.2	79.8			66.9	80.6	87.8	87.2		
≥ 60 min/d	19.5	20.8	20.2			33.1	19.4	12.2	12.8		
**Means of transportation to and from school**				2.287	0.130					11.485	0.009
Passive	37.2	34.5	35.8			63.3	65.8	57.1	60.0		
Active	62.8	65.5	64.2			36.7	34.2	42.9	40.0		
**Time from home to school**				0.266	0.606					84.710	< 0.001
< 10 min/d	48.4	49.1	48.8			35.1	49.1	56.7	65.2		
≥ 10 min/d	51.6	50.9	51.2			64.9	50.9	43.3	34.8		
**Muscle strength training**				23.157	< 0.001					12.248	0.007
< 1 d/wk	90.2	85.8	88.0			91.2	88.0	85.6	84.1		
≥ 5 d/wk	9.8	14.2	12.0			8.8	12.0	14.4	15.9		
**Sedentary Behaviors**											
**Sit and study after school**				22.627	< 0.001					64.211	< 0.001
< 60 min/d	36.2	29.8	33.0			45.3	32.4	25.0	25.6		
≥ 60 min/d	63.8	70.2	67.0			54.7	67.6	75.0	74.4		
**Watch TV**				0.616	0.433					20.842	< 0.001
< 60 min/d	7.1	6.6	6.8			7.1	6.2	8.3	13.7		
≥ 60 min/d	92.9	93.4	93.2			92.9	93.8	91.7	86.3		
**Using a phone or tablet**				3.125	0.077					26.083	< 0.001
< 60 min/d	15.9	14.1	15.0			13.3	14.3	17.3	26.0		
≥ 60 min/d	84.1	85.9	85.0			86.7	85.7	82.7	74.0		

Means of transportation to and from school: passive includes taking a shared vehicle or private car; active includes walking or cycling

*PA* Physical activity

For PA, there was a difference in the proportions of low-intensity PA per day by sex (*χ*^2=^16.058, *P* < 0.001), as well as in the proportions of MVPA per day (*χ*^2=^32.704, *P* < 0.001) and muscle strength training per week (*χ*^2=^23.157, *P* < 0.001). Children and adolescents with different nutritional statuses differed in terms of after school PA, means of transportation to and from school, time from home to school, and muscle strength training (*χ*^2=^94.472, 11.485, 84.710, 12.248, respectively; *P* < 0.05 or 0.001).

For sedentary behaviors, there is a difference in the proportion of sitting to study after school each day by sex (*χ*^2=^22.627, *P* < 0.001). Children and adolescents with different nutritional statuses differed for this variable as well as for watching TV and using a phone or tablet (*χ*^2=^64.211, 20.842, and 26.083, respectively; *P* < 0.001).

### Logistic regression analysis of risk factors among children and adolescents with different nutritional status

**Fig. 3:**
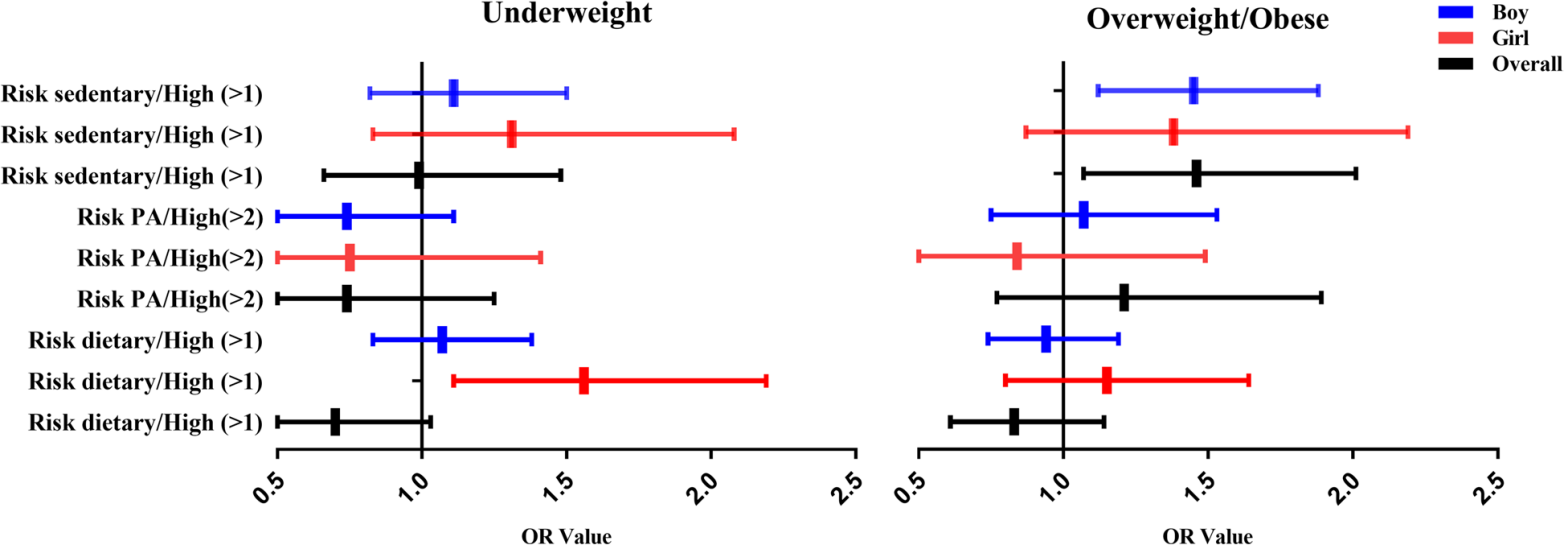
Schematic of adjusted odds ratio (OR) in children and adolescents with different nutritional statuses. Abbreviations: PA, physical activity

According to Table 3, the AOR for risk of being overweight/obese was 1.46 times higher among children with high sedentary risk than that of children with low sedentary risk (95% CI: 1.07–2.01). For girls, the risk for underweight was 1.56 times higher among those with high dietary risk than girls low dietary risk (95% CI: 1.11– 2.19). For all participants, the risk of being overweight/obese was 1.45 times higher among children with high sedentary risk than that for children with low sedentary risk (95% CI: 1.12– 1.88), as shown in Fig. 3.

**Table 3:** Logistic regression analysis of the risk factors among children and adolescents with different nutritional statuses

	**n**	**Normal**	**Underweight**			**Overweight/Obese**		
		**%**	**%**	**COR(95%CI)**	**AOR(95%CI)**	**%**	**COR(95%CI)**	**AOR(95%CI)**
**Boy**								
**Risk dietary**								
Low (≤ 1)	2119	85.8	91.0	1.00	1.00	87.3	1.00	1.00
High (> 1)	321	14.2	9.0	0.62(0.42 ~ 0.90)^*^	0.70(0.47 ~ 1.03)	12.7	0.95(0.71 ~ 1.28)	0.83(0.61 ~ 1.14)
**Risk PA**								
Low (≤ 2)	128	8.7	11.4	1.00	1.00	7.4	1.00	1.00
High(> 2)	1349	91.3	88.6	0.70(0.42 ~ 1.18)	0.74(0.43 ~ 1.25)	92.6	1.25(0.80 ~ 1.95)	1.21(0.77 ~ 1.89)
**Risk sedentary**								
Low (≤ 1)	242	9.0	9.0	1.00	1.00	13.5	1.00	1.00
High (> 1)	2198	91.0	91.0	0.89(0.60 ~ 1.31)	0.99(0.66 ~ 1.48)	86.5	1.58(1.17 ~ 2.14)^**^	1.46(1.07 ~ 2.01)^*^
**Girl**								
**Risk dietary**								
Low (≤ 1)	2033	85.7	82.0	1.00	1.00	82.4	1.00	1.00
High (> 1)	360	14.3	18.0	1.28(0.93 ~ 1.78)	1.56(1.11 ~ 2.19)^*^	17.6	1.23(0.86 ~ 1.75)	1.15(0.80 ~ 1.64)
**Risk PA**								
Low (≤ 2)	113	7.5	9.8	1.00	1.00	9.3	1.00	1.00
High(> 2)	1314	92.5	90.2	0.78(0.41 ~ 1.46)	0.75(0.40 ~ 1.41)	90.7	0.82(0.47 ~ 1.46)	0.84(0.47 ~ 1.49)
**Risk sedentary**								
Low (≤ 1)	182	7.2	8.5	1.00	1.00	10.0	1.00	1.00
High (> 1)	2211	92.8	91.5	1.15(0.73 ~ 1.79)	1.31(0.83 ~ 2.08)	90.0	1.41(0.90 ~ 2.22)	1.38(0.87 ~ 2.19)
**All participants**								
**Risk dietary**								
Low (≤ 1)	4152	85.8	87.0	1.00	1.00	85.7	1.00	1.00
High (> 1)	681	14.2	13.0	0.90(0.70 ~ 1.15)	1.07(0.83 ~ 1.38)	14.3	1.02(0.82 ~ 1.28)	0.94(0.74 ~ 1.19)
**Risk PA**								
Low (≤ 2)	241	8.0	10.7	1.00	1.00	8.0	1.00	1.00
High(> 2)	2663	92.0	89.3	0.73(0.49 ~ 1.08)	0.74(0.49 ~ 1.11)	92.0	1.06(0.75 ~ 1.49)	1.07(0.75 ~ 1.53)
**Risk sedentary**								
Low (≤ 1)	424	8.0	8.8	1.00	1.00	12.4	1.00	1.00
High (> 1)	4409	92.0	91.2	1.00(0.75 ~ 1.34)	1.11(0.82 ~ 1.50)	87.6	1.59(1.24 ~ 2.04)^**^	1.45(1.12 ~ 1.88)^*^

For all participants: adjusted for sex, urban/rural residence, school type, father's education, mother's education, and living on campus. For boys and girls individually: adjusted for urban/rural residence, school type, father's education, mother's education, and living on campus

*PA* Physical activity, *COR* Crude odds ratio, *AOR* Adjusted odds ratio

^*^P < 0.05; ^**^P < 0.01

### Correlation analysis of health behavior risk factors with weight and BMI

Table 4 showed that overall, weight had a significant correlation with PA risk (*r* = 0.076, *P* < 0.01) and sedentary behavior risk (*r* = 0.035, *P* < 0.05). BMI had a significant correlation with PA risk (*r* = 0.064, *P* < 0.01) and sedentary behavior risk (*r* = 0.037, *P* < 0.05). By sex, boys performed significant differences than girls. Correlations were also found for school type.

**Table 4:** Correlations among unhealthy dietary, physical activity, and sedentary behaviors and weight status, by sex and school type

*r*	Total	Sex		School Type	
		**Boy**	**Girl**	**Middle School**	**High School**
***n***	4833	2440	2393	3390	1443
**Weight**					
Risk dietary behavior	0.006	0.001	0.038	0.031	-0.063^*^
Risk PA	0.076^**^	0.125^**^	0.028	0.046^*^	0.053
Risk sedentary behaviors	0.035^*^	0.038	-0.002	0.035^*^	0.057^*^
**BMI**					
Risk dietary behavior	0.009	0.003	0.025	0.027	-0.04
Risk PA	0.064^**^	0.093^**^	0.032	0.045	0.046
Risk sedentary behaviors	0.037^*^	0.050^*^	0.004	0.037^*^	0.051

Data reported was the correlation coefficient, denoted as *r*

*PA* Physical activity, *BMI* Body mass index

^*^P < 0.05; ^**^P < 0.01

## Discussion

In this study, we aimed to assess the prevalence of overweight and obesity and its association with dietary behavior and PA in children and adolescents aged 13–18 years in Xinjiang, China. Our results showed that 13.2% of the study population was underweight, 10.2% was overweight, and 4.7% was obese. Underweight and overnutrition often coexist in low- and middle-income countries (LMICs), which is referred to as the “double burden of malnutrition” [29, 34–37]. A study among 177,325 children and adolescents aged 12–15 years in 58 LMICs showed that 10% of the study population in approximately 30 countries had underweight, compared with 10% with overweight and obesity in 45 countries [38]. The detection rate of overweight (10.2%) and obesity (4.7%) among children and adolescents in our study was lower than that of overweight (14.4%) and obesity (11.9%) among children and adolescents in 2016 and lower than the rate of overweight (12.7%) and obesity (4.9%) among children and adolescents in northeast China in 2018 [22, 39]. Another study reported a rate of overweight (13.81%) and obesity (8.03%) among children aged 6–17 years in China from 2016 to 2018 [40], which was greater than the rates in our study population. The proportion of underweight (13.2%) in our study was slightly higher than reported rates of 11.7% in western China in 2014, 7.9% in children age 7–18 years, 5.47% in Xinjiang in 2016, and 7.4% in China in 2018 [41–43]. Our study was carried out at numerous study sites among different ethnic groups in Xinjiang, and the scope was middle and high school age students. Poor economic development and increased school pressure might cause a change of dietary behavior of children and adolescents in Xinjiang and lead to the increased proportion of underweight. This is also associated with lower PA levels owing to the COVID-19 pandemic, especially among boys, which is in line with the findings of several studies reporting that low PA is associated with underweight in children and adolescents [44–46]. Several studies have reported the rebound rate of malnutrition among children and adolescents in both Guangxi and Wuhan, China [47, 48]. Whether underweight among children 13–18 years old in Xinjiang is common in the development of children and adolescents needs further investigation.

As for dietary behaviors, studies have confirmed that the occurrence of obesity and multiple chronic diseases is closely associated with unhealthy eating behaviors [49]. Consistent with findings regarding a low-fat diet among children aged 6–18 years [12], 91.7% of underweight children aged 13–18 years in Xinjiang, China consume dairy or soy milk ≥ 1 d/week. Every 100 g of whole milk provides 64 kcal of energy and 3.5 g of protein. Therefore, milk makes an important contribution to the nutritional intake of children and adolescents [30]. Xinjiang is an economically underdeveloped region, with economic resources mainly involving agriculture and animal husbandry. Thus, the annual per capita consumption of milk in Xinjiang is higher than the national level. A larger proportion of children and adolescents in Xinjiang consume milk compared with other regions that have low dairy intake, and high milk intake may be an influencing factor for underweight. There are few investigations of underweight status in the study region. Greater attention is needed regarding underweight and healthy brain development in children and adolescents in this area [28].

Excessive consumption of sugary drinks can increase the risk of overweight or obesity in children and adolescents, and consumption of sweetened drinks has become a common behavior in these age groups globally. With the world's overweight and obese population continuing to increase, in 2015, the WHO called for a reduction in sugar intake worldwide [50]. In a study in 2013 among 53,000 Chinese children and adolescents aged 6–17 years, 66.6% of participants consumed sugar-sweetened beverages and 9.6% reported consuming more than seven sugar-sweetened beverage per week [30]. We found that 81.1% and 85.0% of overweight and obese children and adolescents, respectively, consumed sugar-sweetened drinks daily over 1 time, which was higher than the proportions in the normal and underweight groups. Our study confirmed that excessive consumption of sugary drinks is a risk factor for overweight and obesity among children and adolescents, which is consistent with the conclusions of other studies [51–53]. In 2019, the per capita sugar consumption in Xinjiang Uygur Autonomous Region was 1.8 kg, much higher than China's per capita consumption of 1.3 kg and ranking third in the country [14]. Sugar intake in Xinjiang is high, among which children and adolescents might be important groups with high sugar intake. Schools, families, and society should take measures to reduce sugar intake in the population.

There is a positive correlation between PA and energy expenditure [54, 55]. In terms of MVPA, this study showed that 92.9% of children and adolescents aged 13–18 years in Xinjiang had MVPA time of 60 min/d at school, and participants met the recommended MVPA activity standard of the WHO and for Chinese children and adolescents. The results of this study differed from those previous studies in China and abroad. In 2016, Roman-Vinas used acceleration sensors and found that only 15% of children and adolescents objectively measured in Tianjin, China met the daily MVPA recommendations [56]. In 2016, only around 30% of children and adolescents in China met the recommended MVPA volume. Middle and high school students were more likely to meet the recommended MVPA than primary school students [31]. Physical inactivity was positively associated with socioeconomic level [57]. Parental educational and profession levels have been also associated with adolescents’ sedentary behaviour [58]. The proportion of Chinese children and adolescents meeting MVPA between 2016 and 2018 did not exceed 40% [40]. Regarding the reason for these differences, differences exist in the questionnaires or instruments used for PA assessment among different studies. This may also be related to different survey populations, ages, regional economic development levels, school types, and levels of academic pressure. Our study also showed that 20.2% of students engaged in 60 min/d PA daily after school, which is consistent with studies reporting that Chinese children and adolescents engaged in more MVPA on campus than off campus [32, 33]. In terms of sex, we found that the proportion of boys participating in 60 min/d MVPA per day was 95%, significantly higher than the 90.7% of girls. Boys were also more likely to meet MVPA recommendations than girls, which is consistent with results obtained in China and abroad [31, 59–62].

It is well known that economic and technological advancements have increased screen time among young people [63–65]. In China, 41.5% of teens spend 2 h per day using a screen on weekends [66]. The influencing factors of body function include sedentary behaviors in addition to dietary behaviors and PA. The analysis of sedentary behaviors in this study included three categories: sitting to study after school, watching TV, and using a phone or tablet. Logistic regression analysis also showed that children and adolescents with high sedentary risk had a 1.59-fold and 1.45-fold greater risk of developing overweight and obesity, respectively, compared with their counterparts who had low sedentary risk. Additionally, correlation analysis indicated that the risk of sedentary behavior showed a positive correlation with weight (*r* = 0.035) and BMI (*r* = 0.037). The conclusions of this study are consistent with the results of previous related studies. Reducing stationary behavior is an important means to prevent and treat overweight and obesity among children and adolescents [21, 67–70]. Because screen-based behavior is often accompanied by decreased PA, snack intake, and consumption of sugary drinks, which affect the energy balance, this can lead to the occurrence of overweight or obesity. Therefore, another benefit of reducing static behavior is a reduction in calorie intake [71]. Current evidence suggests that screen media exposure leads to obesity in children and adolescents through increased eating while viewing; exposure to high-calorie, low-nutrient food and beverage marketing that influences children’s preferences, purchase requests, consumption habits; and reduced sleep duration [23]. Greater participation in PA and limiting screen time are important interventions to prevent obesity and regulate physical health.

There are several advantages of this study. First, ours is the first extensive survey of nutritional status, dietary behavior, and PA among children and adolescents in Xinjiang, China. Second, the study region is a multi-ethnic area with a unique geographic environment and varied eating and lifestyle habits. Our survey of children and teenagers in this region has distinct regional characteristics.Third, this study includes a large, diverse sample of students recruited using multistage and stratified cluster random sampling that provides an adequate representation of Xinjiang children and adolescents. We adjusted for several confounders that may influence the studied associations. Our study also has some limitations. First, we used self-report questionnaires, which can be affected by recall bias and may deviate from the actual values, for example, MVPA may be somewhat overestimated. Objective measurement of PA is challenging and costly in a large sample, necessitating the use of specialized equipment and technical staff. Second, the sample size and number of dietary behaviors investigated in this study may be insufficient. Improved future studies are needed to achieve more accurate results.Third, such assessments may not accurately reflect true nutritional status and may underestimate the magnitude of the observed associations. Finally, this study is a cross-sectional study, which can only understand the association between different factors, but cannot understand the causal relationship between them.

## Conclusions

The detection rate of underweight was high among children aged 13–18 years in Xinjiang, China but low for overweight and obesity, compared with the whole country. Sedentary behavior is an important factor impacting the occurrence of overweight and obesity in children and adolescents in Xinjiang.This test is the first step in understanding the current status of overweight and obesity, physical activity and nutritional status among children and adolescents of various ethnic groups in Xinjiang, and has obtained extensive available data for China. Taking this as an opportunity, continued adherence to monitoring of physical activity will help guide the development of policies and programs to increase the activity levels of children and adolescents in Xinjiang and reduce the burden of NCDs.

## Data Availability

The datasets used during the current study cannot be made publicly available as per ethics approval at Chizhou University. Readers can obtain them from the corresponding author on reasonable request.
